# Compact biologically inspired camera with computational compound eye

**DOI:** 10.1515/nanoph-2023-0782

**Published:** 2024-04-23

**Authors:** Shu-Bin Liu, Xu-Ning Liu, Wei-Jie Fan, Meng-Xuan Zhang, Lei Li

**Affiliations:** School of Electronics and Information Engineering, 12530 Sichuan University, Chengdu 610065, China; Faculty of Science, The University of Melbourne, Victoria, 3010, Australia

**Keywords:** compound eye, imaging system, deep learning, integrated optics

## Abstract

The growing interests have been witnessed in the evolution and improvement of artificial compound eyes (CE) inspired by arthropods. However, the existing CE cameras are suffering from a defocusing problem due to the incompatibility with commercial CMOS cameras. Inspired by the CEs of South American Shrimps, we report a compact biologically inspired camera that enables wide-field-of-view (FOV), high-resolution imaging and sensitive 3D moving trajectory reconstruction. To overcome the defocusing problem, a deep learning architecture with distance regulation is proposed to achieve wide-range-clear imaging, without any hardware or complex front-end design, which greatly reduces system complexity and size. The architecture is composed of a variant of Unet and Pyramid-multi-scale attention, with designed short, middle and long distance regulation. Compared to the current competitive well-known models, our method is at least 2 dB ahead. Here we describe the high-resolution computational-CE camera with 271 ommatidia, with a weight of 5.4 g an area of 3 × 3 cm^2^ and 5-mm thickness, which achieves compatibility and integration of CE with commercial CMOS. The experimental result illustrates this computational-CE camera has competitive advantages in enhanced resolution and sensitive 3D live moving trajectory reconstruction. The compact camera has promising applications in nano-optics fields such as medical endoscopy, panoramic imaging and vision robotics.

## 1 Introduction

Over one billion years of evolution, arthropods have developed sophisticated compound eyes (CEs) with extraordinary vision, and the increasing interest in CEs has been observed in their evolution and improvement. Natural insect CEs are natural imaging systems with complex-exceptional capabilities, which include numerous closely distributed ommatidia, which enable to inspire the development of artificial CE. CEs have unique advantages in integration, wide FOV, distortion-free imaging, and sensitive motion tracking ability [1], [2], [3], which promotes the applications in medical endoscopy, panorama, robot vision and micro navigation [4], [5], [6], [7], [8], [9], [10]. In the past decade, great efforts have been devoted to the development of artificial CEs, including macroscopic-array systems and integrated CE. As some typical examples in macroscopic-array-systems field, AWARE-2 [1] and AWARE 40 [11] cameras use array cameras to capture large-scale photography. Moreover, RUSH [12] with 35 CMOSs is proposed to achieve cm-scale FOV and μm-level resolution. DLBP array camera [13] is proposed to achieve panorama and computational zoom imaging. The existing array cameras are suffering from large size and complex design, however, the integrated CEs pave the way for camera integration. Planar CEs were first implemented by combining a microlens array (MLA) with commercial CMOS, whose FOV is relatively small [14]. To enable wide FOV, curved CEs have been successfully fabricated using advanced special surface design and micro-nano fabrication technology [2], [15], [16], [17], [18], [19], [20], [21], [22], [23]. Typically, a CE camera is prepared with an enhanced FOV in the whole directions [17]. A CE camera is proposed with a 14.72-mm size and wide FOV [18]. A curved CE camera is fabricated using specific-complex design and fabrication process [23]. Nevertheless, further integration and enhanced-resolution of the whole CE system becomes significant due to the incompatibility of complex CE and CMOS. To the best of our knowledge, such a compact CE camera without special front-end design, has never been achieved.

In this work, to overcome the defocusing problem, a novel deep learning architecture with distance regulation is proposed to achieve wide-range-clear imaging, without any hardware or specialized front-end design, which greatly reduces system complexity and size. Based on the proposed architecture, a compact computational-CE camera is reported, which enables wide-FOV, high-resolution imaging and sensitive 3D moving trajectory reconstruction, with only a weight of 5.4 g, a 3 × 3-cm^2^ area and 5-mm thickness.

## 2 Results

### 2.1 Principle and concept

**Figure 1:**
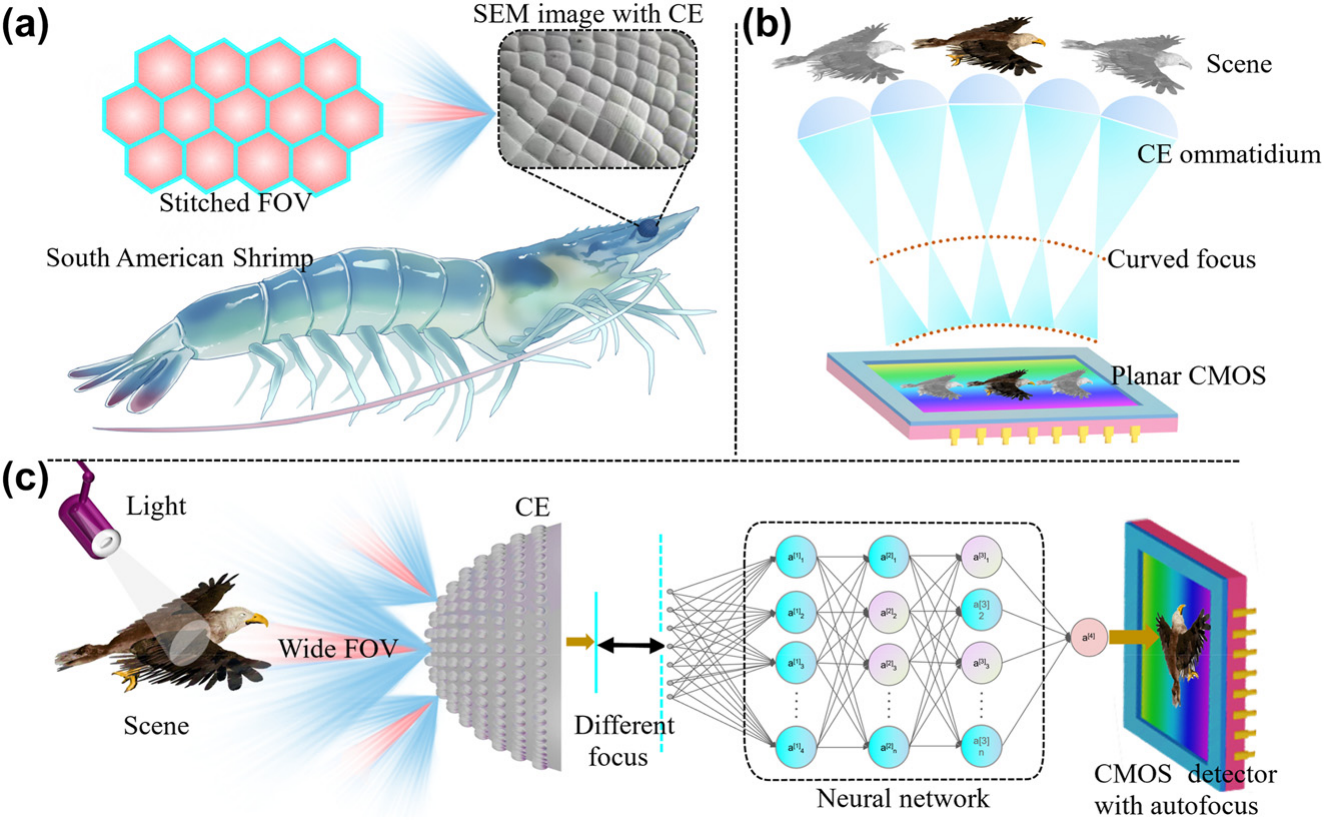
Principle and concept of proposed computational-CE camera. (a) CE of the South American Shrimp. (b) Defocusing principles of curved CE with planar CMOS. (c) Principle of our fabricated computational-CE camera.

Natural Penaeus under a complex environment, with a well-developed visual system densely covered with ommatidia, inspired this work. As illustrated in Figure 1(a), the CE of South American Shrimp is composed of a series of ommatidia under a scanning electron microscope (SEM), which is regarded as large FOV. Artificial CEs perfectly inherit the advantages of natural CEs, however, the direct integration of traditional CEs with planar CMOS will result in defocusing blurring and inability to achieve perfect imaging due to the curved focusing, as illustrated in Figure 1(b). The concept of computational-CE camera is inspired by above inspiration. In Figure 1(c), after passing through a CE, incident light will be focused onto different planes, which results in blurred imaging with an inhomogeneous resolution. To address the challenges of incompatibility between traditional CEs and commercial CMOS, here this work cleverly combines a proposed multi-branch model with CE camera to resolve complete-focused imaging with uniform resolution from degraded ones, without any additional hardware and complex front-end design.

## 3 Methods

### 3.1 PDMS CE fabrication methods

**Figure 2:**
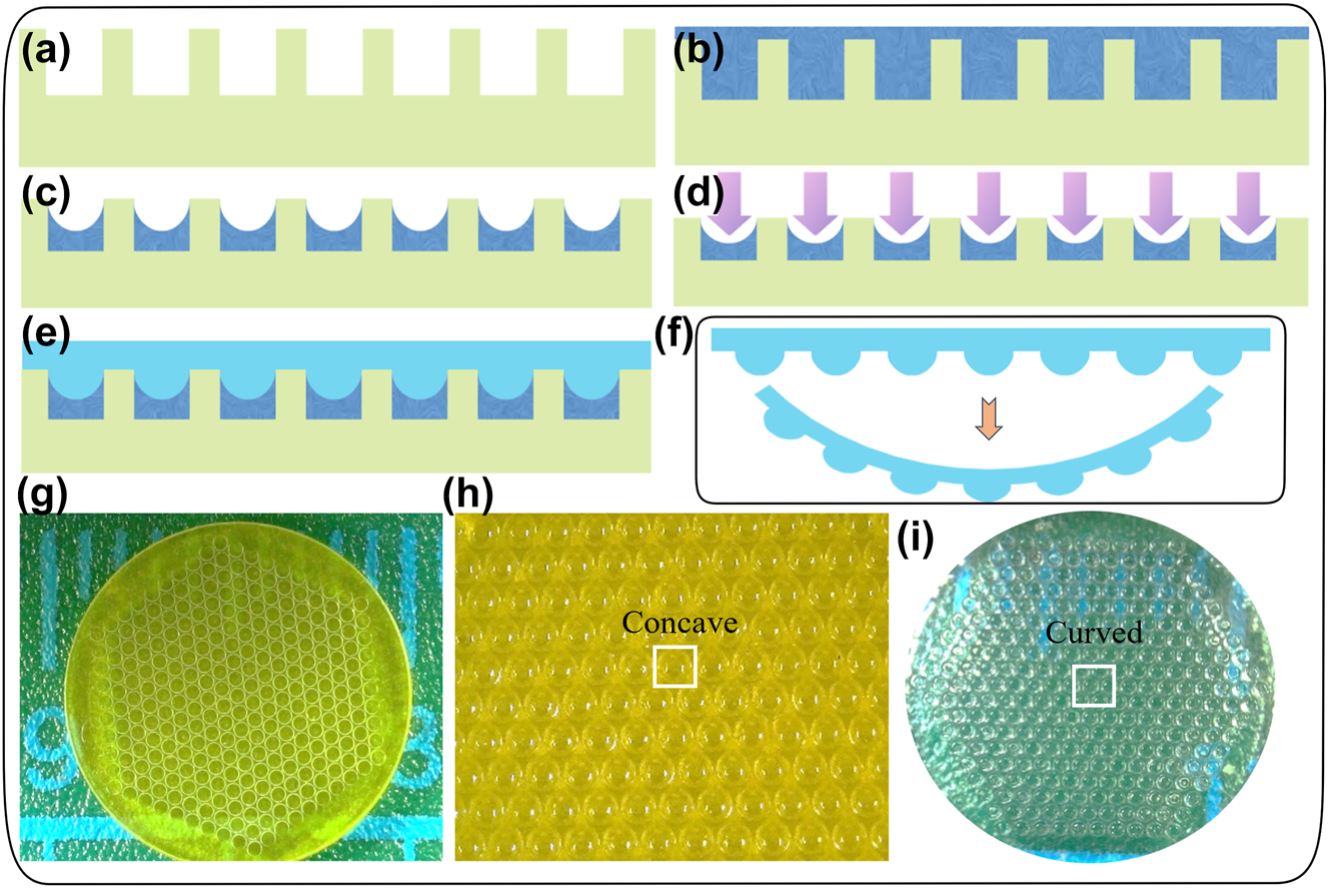
Fabrication of PDMS CE. (a) 3D printing substrate using nanoArch^®^ S140. (b) Microholes filled with SU-8. (c) Uncured mold. (d) Fabricated cured mold. (e) PDMS fabrication. (f) Cured PDMS fabrication. (g) Physical images of the substrate. (h) Physical images of the mold. (i) Physical images of the curved PDMS.

The fabrication of polydimethylsiloxane (PDMS) CE involves six steps, as shown in Figure 2(a)–(f). Firstly, the substrate is fabricated by a nanoArch^®^ S140 3D printing system with 25-um precision (BMF Precision Tech Inc. Shenzhen, China), and the photosensitive resin UTL is used as the printing material. Secondly, the microholes of the substrate are filled with photoresist (SU-8). Then, it is degassed for 1 h in a vacuum machine (FUJIWARA-550D) for degassing and the bubbles floating on the photoresist surface are removed with a tool. Then it was placed in KW-4A coater at a speed of 3000 r/s for 120 s for homogenizing of the glue, thereby forming a preliminary microlens array (MLA) mold. The mold is then placed on a heating platform (CHEMAT-MODELKW-4AH) for initial curing for 15 s, and then exposed to ultraviolet light to final cure, completing the mold fabrication. Then, the PDMS liquid is fabricated by mixing DowCorning’s Sylgard 184 in a ratio of 10:1. The mixture is degassed using a vacuum machine and poured into the mold. Subsequently, it is heat-cured at 75–80 °C for 1.5 h, resulting in the formation of a planar PDMS film with the MLA. Finally, the planar PDMS film with the MLA is pushed to a heated shell slowly from the surface without the MLA. After a few minutes when it dropped to room temperature, the curved shell was torn off with the MLA.

The physical images of the substrate, mold, and curved PDMS filmed with the MLA are shown in Figure 2(g)–(i). In Figure 2(g), the total number of holes in the substrate is 271, and the diameter of each hole is 400 μm, and the diameter of the whole substrate is 9 mm. In Figure 2(i), the diameter of the entire PDMS compound eye is 8 mm, weighs 0.046 g, and the MLA has 271 ommatidia, each of which has a diameter of 400 μm and a refractive index of 1.4.

### 3.2 Imaging pipeline and designed network architecture

**Figure 3:**
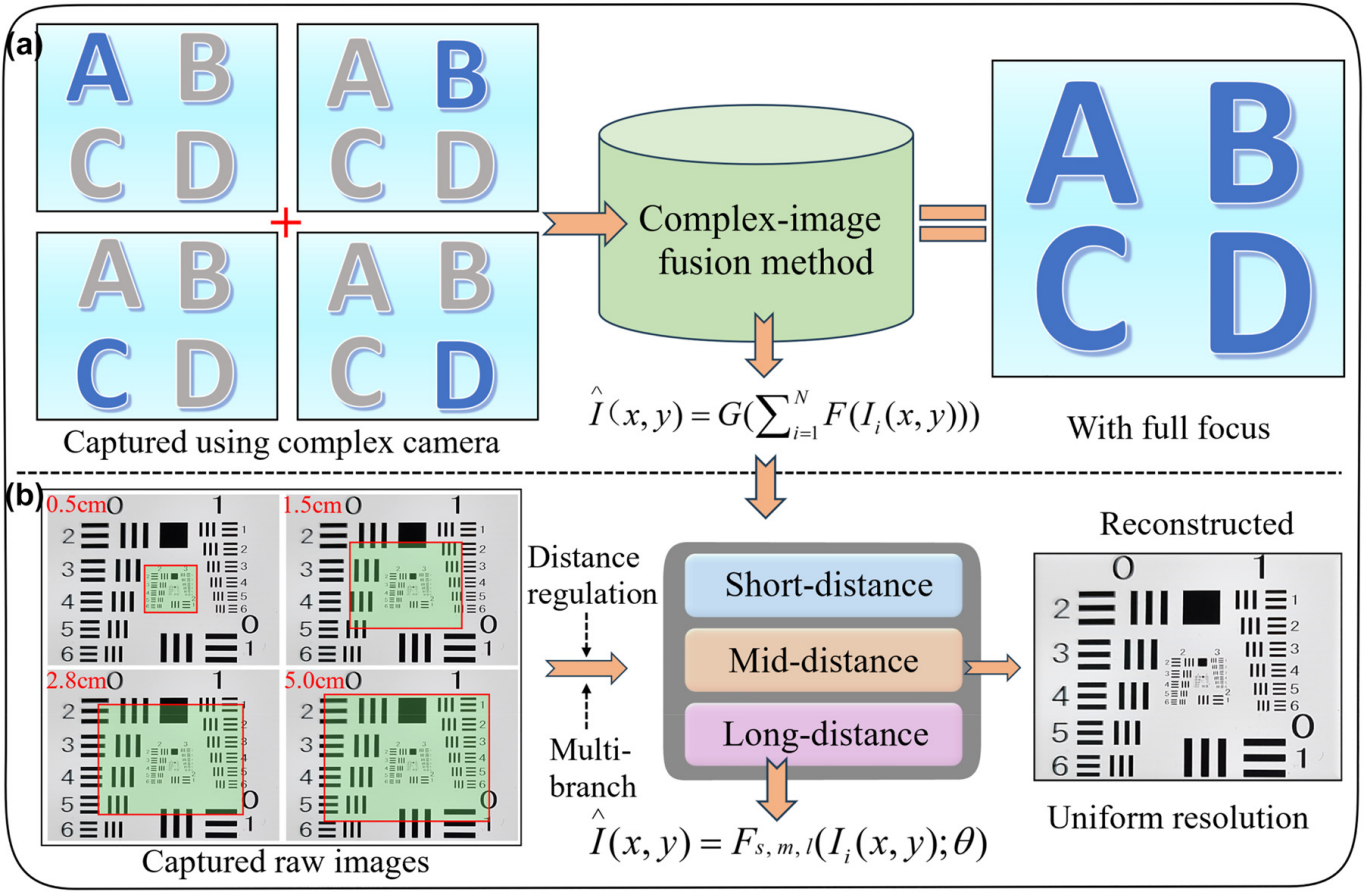
Comparisons of traditional imaging strategy with ours. (a) Traditional imaging pipeline, where blue letters represent focusing state, while gray letters represent defocusing state. (b) Our proposed imaging strategy with distance regulation, where green represents defocusing imaging.

The traditional imaging pipelines are typically limited to complex systems and methods, previously proposed CE cannot usually account for single-frame imaging. As illustrated in Figure 3(a), the captured raw-defocused images in various distances using complex-designed CE, are composited as an image with full focus using complex-image fusion method, such as Laplace pyramid fusion algorithm [24]. In general, the calculated process can be described as:

(1) $$\overset{\wedge }{I}\left.〈x,y\right)=G\left(\overset{N}{\underset{i=1}{\sum }}F\left(I_{i}\left(x,y\right)\right)\right)$$

where $\overset{\wedge }{I}\left(x,y\right)$ and *I*_*i*_(*x*, *y*) represent the composited full-focus image and captured raw-defocused image, respectively. *F*(·) represents the operation for captured raw-defocused image, and *G*(·) represents the complex-image fusion method. The fidelity of the obtained information can be reduced due to defocusing blurring caused by different distances. Furthermore, it can be foreseen that the blurring caused by different distances varies. To address these challenges, here a novel multi-branch imaging strategy with distance regulation for CE camera is proposed, as illustrated in Figure 3(b). The captured raw-defocused images in various distances are recovered using the multi-branch model with distance regulation, including short-distance, mid-distance and long-distance branch. Using the end-to-end model instead of asynchronous captures only, the final-composited image $\overset{\wedge }{I}\left(x,y\right)$ for uniform-resolution image can be described as:

(2) $$\overset{\wedge }{I}\left(x,y\right)=Fs,m,l\left(I_{i}\left(x,y\right);\theta \right)$$

where *I*_*i*_(*x*, *y*) represents the captured raw-defocused image, *F*_*s*,*m*,*l*_ represents the deconvolution operation for the end-to-end model in short, middle and long distance, and *θ* represents the parameters in the model. This integrated imaging strategy avoids complex design and processing, which considerably mitigates blur due to the imperfect imaging and diffraction of the imaging system, in tandem with possible sensor noise.

**Figure 4:**
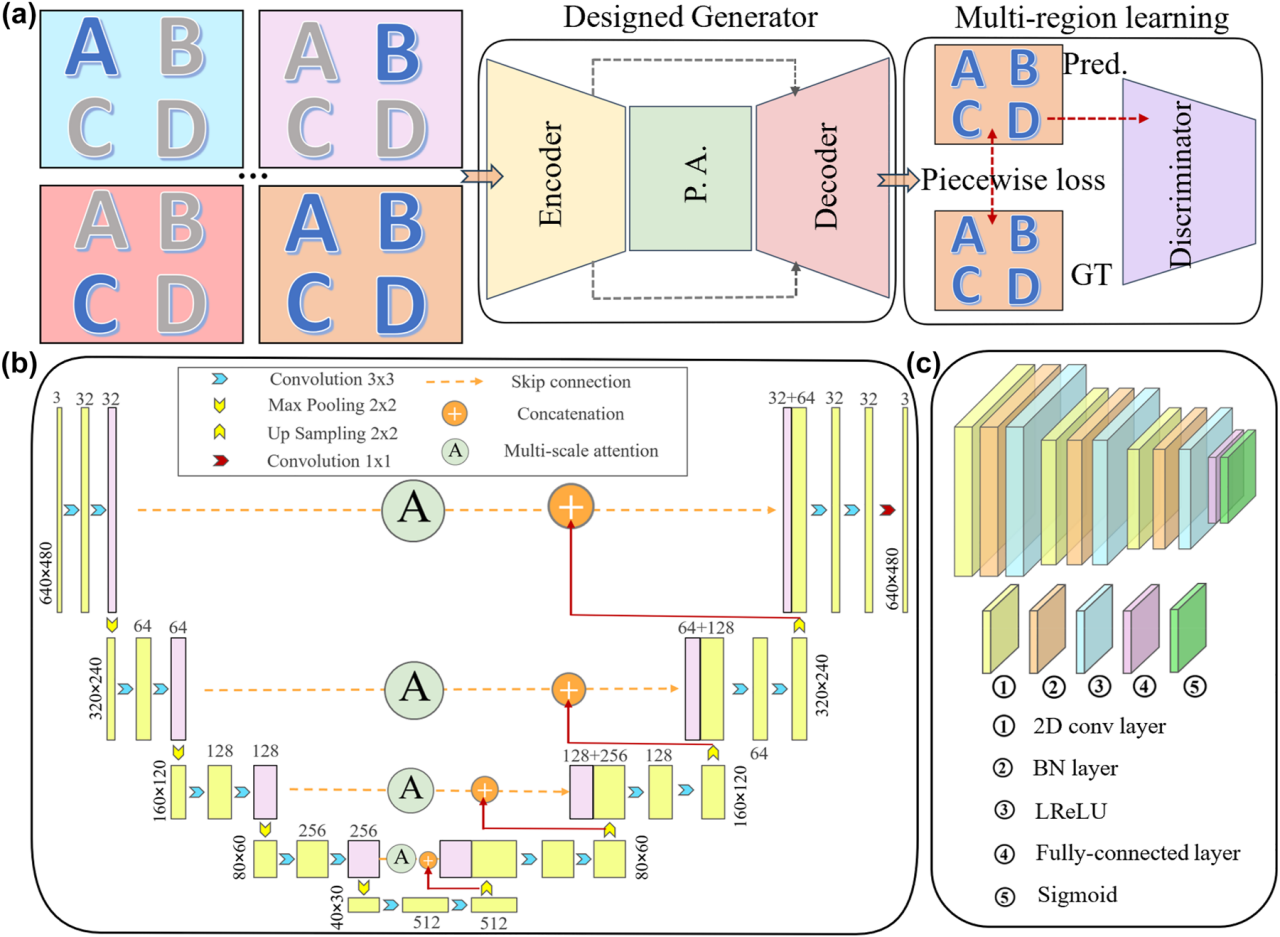
Designed overall architecture with distance regulation for fabricated camera. (a) Overall architecture for designed network. (b) Designed generator. (c) Designed discriminator.

The detailed architecture for the multi-branch model in Figure 3(b) is illustrated in Figure 4. Figure 4(a) illustrates the multi-branch model is mainly consisting of a designed generator and discriminator. Considering the ambiguity caused by different distances in the real world, a multi-branch strategy based on distance regulation is proposed. For the captured dataset, a slide is specially arranged to ensure that the low resolution (LR) raw images {*I*_*i*_(*x*, *y*)} captured by the CE camera are based on different distances. In order to avoid external factors such as jitter as much as possible, we choose a commercial electric zoom lens to capture high-resolution Ground Truth (GT) images {*I*_real_^GT^}. In theory, according to the arrangement of our PDMS ommatidium array, it can be roughly divided into 10 rings, and the ommatidia on the same ring have the same focal length, object distance, and other characteristics. However, considering that the depth of focus has a certain adjustment range, the imaging range of each ring can take into account a certain area, and it is reasonable to choose the three distances of long, middle, and short distances for adjustment. Here the raw image clusters subject to distance are trained, including short-distance clusters, mid-distance clusters and long-distance clusters. For example, for short-distance clusters, given a set of LR raw images {*I*_*i*_(*x*, *y*)} and corresponding GT images {*I*_real_^GT^}, the same applies to the other two. The designed generator is a variant of Unet structure, which consists of an encoder, multi-scale attention and decoder. Due to regional variations in CE imaging, a piecewise loss is considered, to strengthen the recovery of texture details while reducing the impact of smooth areas. In this work, 700 raw image pairs captured using CE camera and high-performance camera, are trained using our multi-branch model and piecewise loss function. In each branch of the reconstruction model, we attempt to adopt the joint training of adversarial loss [25], perceptual loss [26] and L1 [27] to recover degraded images robustly at different distances.

### 3.3 Adversarial loss

Adversarial Loss function is used to guide the generator to generate realistic images, here we apply Wasserstein GAN to minimize the Wasserstein distance between predictions and GT for a robust training process. The adversarial loss function can be expressed as:

(3) $$\begin{array}{ll} \text{Advloss} & =E_{\overset{\wedge }{x}∼\text{Pg}}\left[D\left(\overset{\wedge }{x}\right)\right]-E_{x∼Pr}\left[D\left(x\right)\right]\\ & \;+\lambda E_{\overset{\wedge }{x}∼P\overset{\wedge }{x}}\left[{\left({\left\|\nabla \overset{\wedge }{x}D\left(\overset{\wedge }{x}\right)\right\|}_{2}-1\right)}^{2}\right] \end{array}$$

where Pr represents the GT data {*I*_real_^GT^}, and Pg represents the generated data { $\overset{\wedge }{I}\left(x,y\right)$ }.

### 3.4 Perceptual loss

Perceptual loss is used to improve the visual quality of generated images $\overset{\wedge }{I}\left(x,y\right)$ , whose goal is to minimize the distance between the generated image and GT image in the feature space. The expression can be described as:

(4) $$\begin{array}{ll} \text{Perceploss} & =\frac{1}{N}\overset{N}{\underset{i=1}{\sum }}\left(Fs,m,l\left(\overset{\wedge }{I}\left(x,y\right)\right)\right.\\ & \;{\left.-Fs,m,l\left({I_{\text{real}}}^{\text{GT}}\right)\right)}^{2} \end{array}$$

### 3.5 L1 loss

L1 loss function is used to reconstruct pixel level similarity, which is equal to calculate the differences between GT images *I*_real_^GT^ and generated images $\overset{\wedge }{I}\left(x,y\right)$ . The expression can be described as:

(5) $$\text{L}1\text{loss}=\frac{1}{N}\overset{N}{\underset{i=1}{\sum }}\left|{I_{\text{real}}}^{\text{GT}}-\overset{\wedge }{I}\left(x,y\right)\right|$$

Hence, the total loss can be calculated using the combination of formulas (3)–(5):

(6) Totloss=Advloss+Perceploss+L1loss

Using the piecewise loss, all parameters are optimized using the optimization functions.

Figure 4(b) illustrates the overall architecture designed generator, including variant for a combination of Unet and Pyramid-multi-scale attention [28], which enhances the reconstruction for multi-scale texture information. Also, the details of discriminator are illustrated in Figure 4(c), which covers 2D convolution layer, BatchNom layer, LReLU function, fully-connected layer and Sigmoid function. Here the generator is to generate realistic images to deceive the discriminator, and the discriminator is to distinguish the authenticity of an image. Our work is performed on a PC platform (Intel Core i9-10850K CPU @3.6 GHz + GTX3060Ti) equipped with Windows 10 operating system.

### 3.6 System

**Figure 5:**
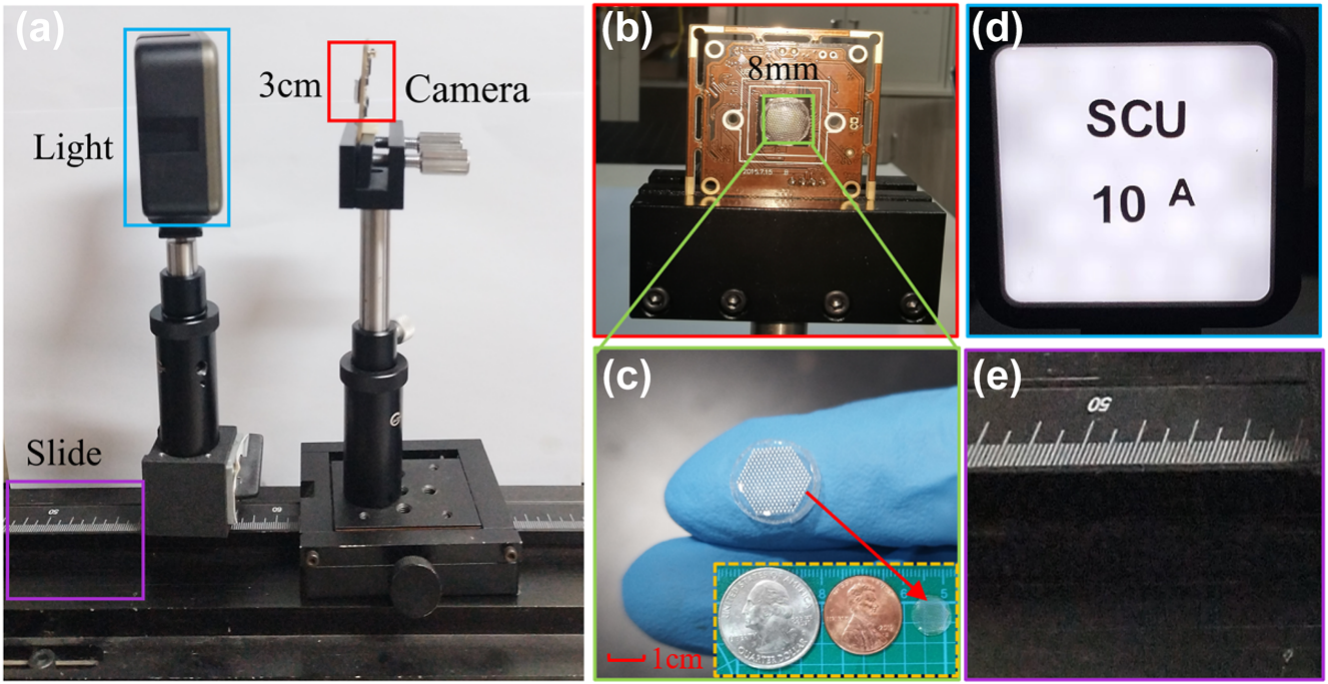
Experimental setup with compact computational-CE camera. (a) System setup. (b) Computational-CE camera. (c) Comparisons of PDMS CE with coins. (d) Light source. (e) Slide.

The computational-CE camera is a highly integrated system, which has a competitive advantage in scale, weight, and cost. As illustrated in Figure 5(a), the light source and the computational-CE camera are attached to a graduated slide. The computational-CE camera is integrated on a 3 × 3 cm^2^ circuit board, only with a 5-mm thickness and a weight of 5.4 g, which significantly simplifies system size and complexity. The CE is with a 4-mm radius, as illustrated in Figure 5(b). As illustrated in Figure 5(c), the comparison result demonstrates that the size of CE has an integrated advantage comparing with 1-cent and 25-cent coins. The light source adopts LED illustrated in Figure 5(d) to irradiate the sample, and the distance can be adjusted according to the scale using the slide in Figure 5(e). Here we describe the computational-CE camera with 271 ommatidia, only with a weight of 5.4 g, an area of 3 × 3 cm^2^ and 5-mm thickness.

### 3.7 Enhanced-contrast reconstruction

**Figure 6:**
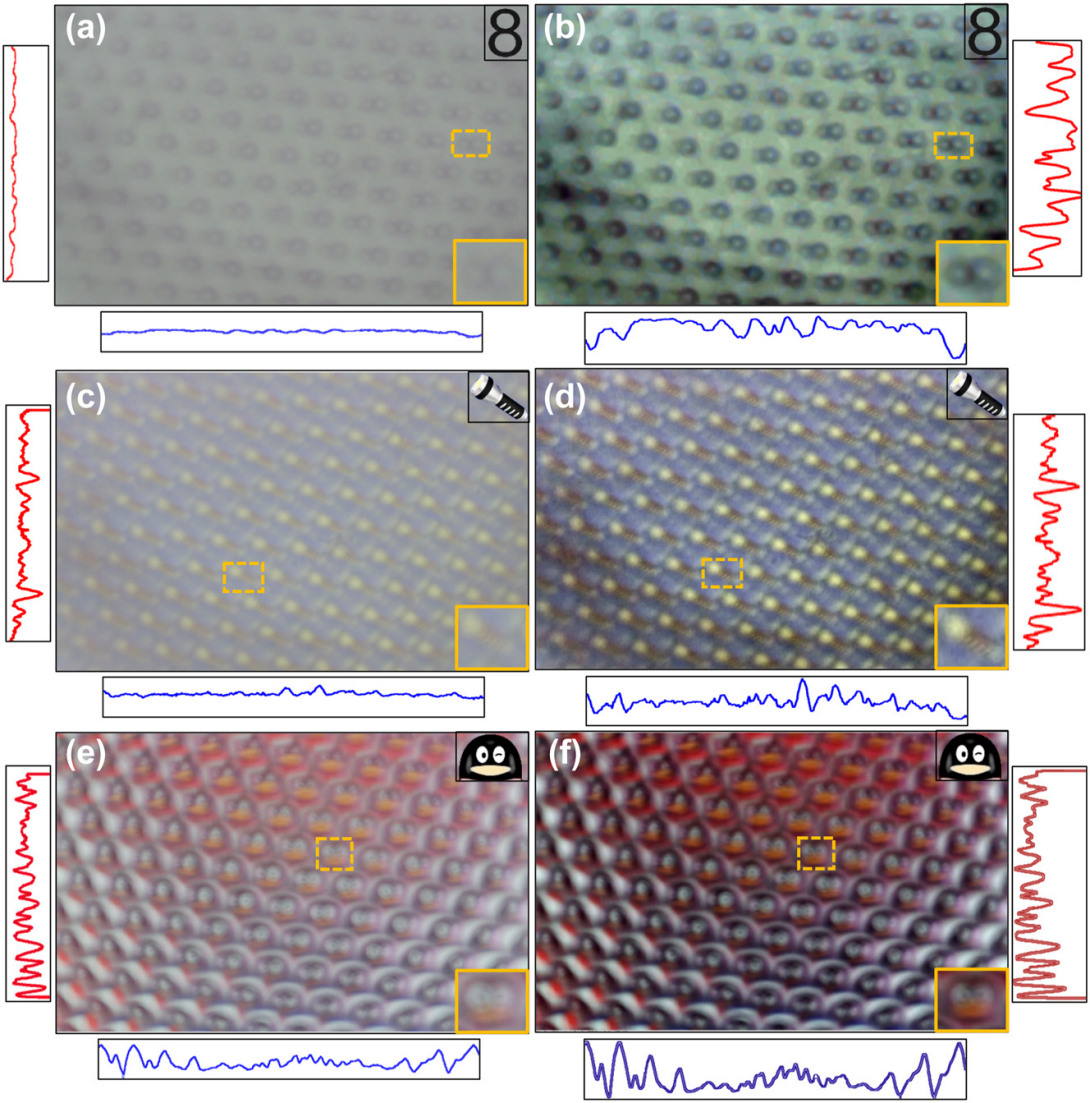
Imaging result comparisons and analysis for our proposed compact computational-CE camera. (a, c, e) Left: the traditional CE camera. (b, d, f) Right: our fabricated CE camera.

Traditional CE cameras perform poorly in the face of natural scene in visible light, which inevitably results in defocusing blur due to the imperfect imaging. However, the experimental result in Figure 6 shows the competitive advantage comparing with the traditional CE camera. The sample I for an Arabic numeral “8” located in the upper right corner is with a defocusing blur due to imperfect imaging in Figure 6(a). However, is recovered effectively in Figure 6(b), and the labeled-yellow region represents the reconstruction detail. It is worth noting that the red and blue line chart are the normalized intensities that vary with the coordinates for the corresponding image, and the positions are the middle lines of the corresponding image (the same follow-up). The sample II for a painted flashlight is reconstructed incompletely in Figure 6(c), such as how many stripes are exactly located on the flashlight. The labeled-yellow in Figure 6(d) illustrates 4 stripes are located on it, which is unreadable from Figure 6(c). The sample III for a painted penguin face is with a blurred reconstruction, as illustrated in Figure 6(e), which cannot be distinguished from the background considerably. As a comparison in Figure 6(f), the reconstructed face using our fabricated camera with the deep learning model is successfully distinguished. For the reconstructed analysis, the normalized intensity statistics varied with coordinate showing greater contrast ratio. Using the computational-CE camera strategy, the experimental result demonstrates significant enhancements in contrast and sharpness.

**Figure 7:**
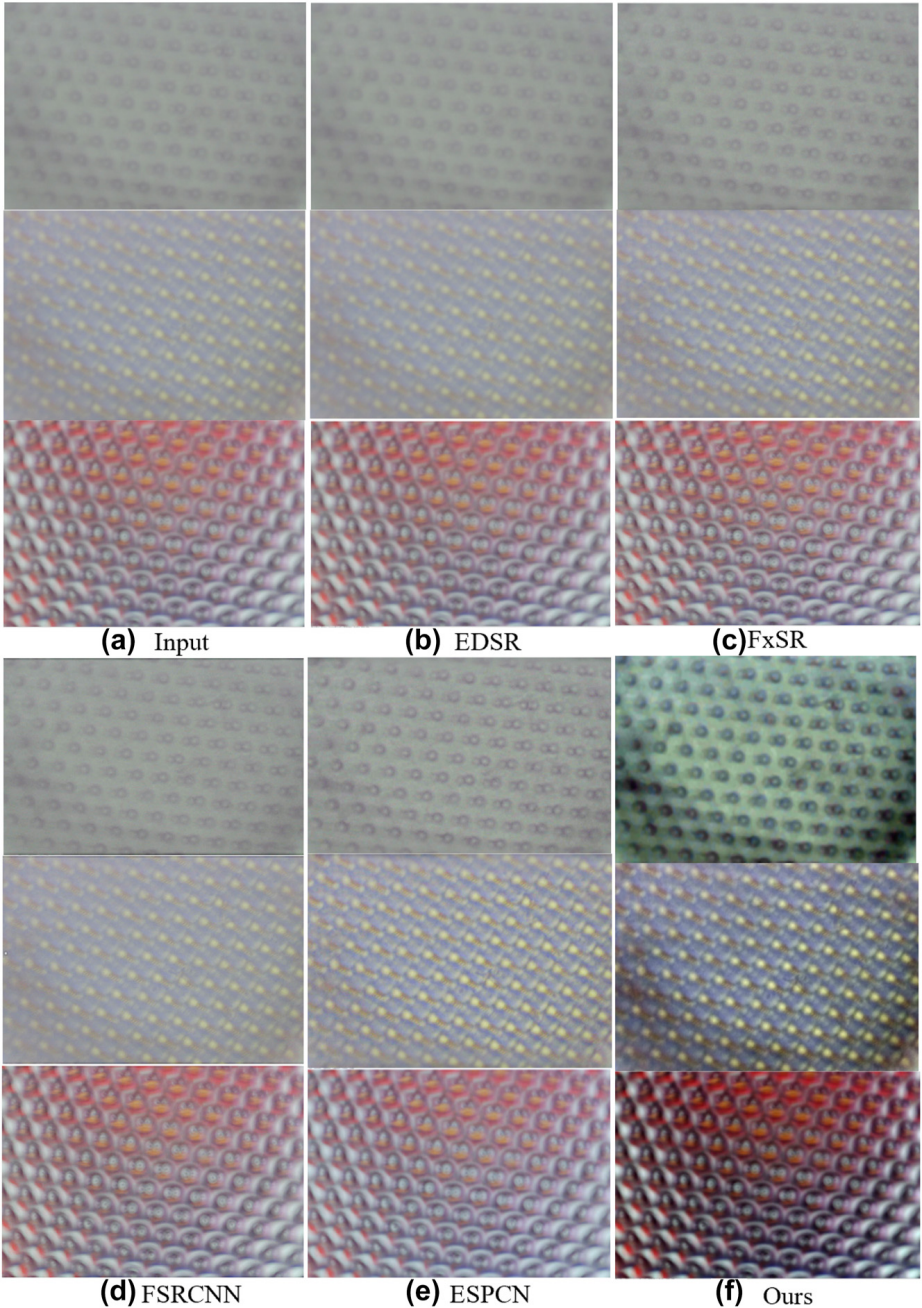
Comparison results with conventional SR reconstruction methods. (a) Input. (b) EDSR. (c) FxSR. (d) FSRCNN. (e) ESPCN. (f) Ours.

In real-world super-resolution reconstruction tasks, efficiency and quality are the golden rules for measuring models. There are classical models that are robust in terms of reconstruction quality and detail, which are often served as the comparison model, such as single image super-resolution (EDSR) [29], flexible style image super-resolution (FxSR) [30], fast super-resolution convolutional neural network (FSRCNN) [31] and efficient sub-pixel convolutional neural network (ESPCN) [32]. As illustrated in Figure 7, we compared the reconstruction results of our model with the world-famous SR models, from which it can be seen that our reconstruction effect is ahead of other models. From the example, the advantage is to resist blurred phenomenon, and the ability to describe texture details is more prominent due to the addition of multiscale attention mechanism. And a quantitative analysis was compared as shown in Table 1. The first column of images represents the input images of different examples, while the 2–6 columns of images represent the results of EDSR, FxSR, FSRCNN, ESPCN and our reconstruction model processing for different examples, respectively. The results of the comparison can be concluded that our reconstruction method is ahead of the reconstruction models in recent years in terms of imaging quality, human eye vision and efficiency. Our reconstruction method has a competitive advantage in quantitative scoring comparing with other SR reconstructed methods.

**Table 1:** Quantitative analysis of comparative results.

Test dataset	Upscaling factor	Ours			EDSR			FxSR		
		PSNR	SSIM	Time	PSNR	SSIM	Time	PSNR	SSIM	Time
Raw-cap	×4	31.21	0.863	0.11	29.11	0.814	8.29	30.76	0.820	0.35
City-100		30.76	0.890	0.11	29.25	0.828	8.78	30.33	0.826	0.33
**Test dataset**	**Upscaling factor**	**FSRCNN**			**ESPCN**					
		**PSNR**	**SSIM**	**Time**	**PSNR**	**SSIM**	**Time**	
Raw-cap	×4	28.47	0.732	0.15	29.48	0.828	0.21	PSNR: dB		
City-100		28.16	0.787	0.18	29.66	0.795	0.19	Time: second		

### 3.8 Moving trajectory reconstruction

**Figure 8:**
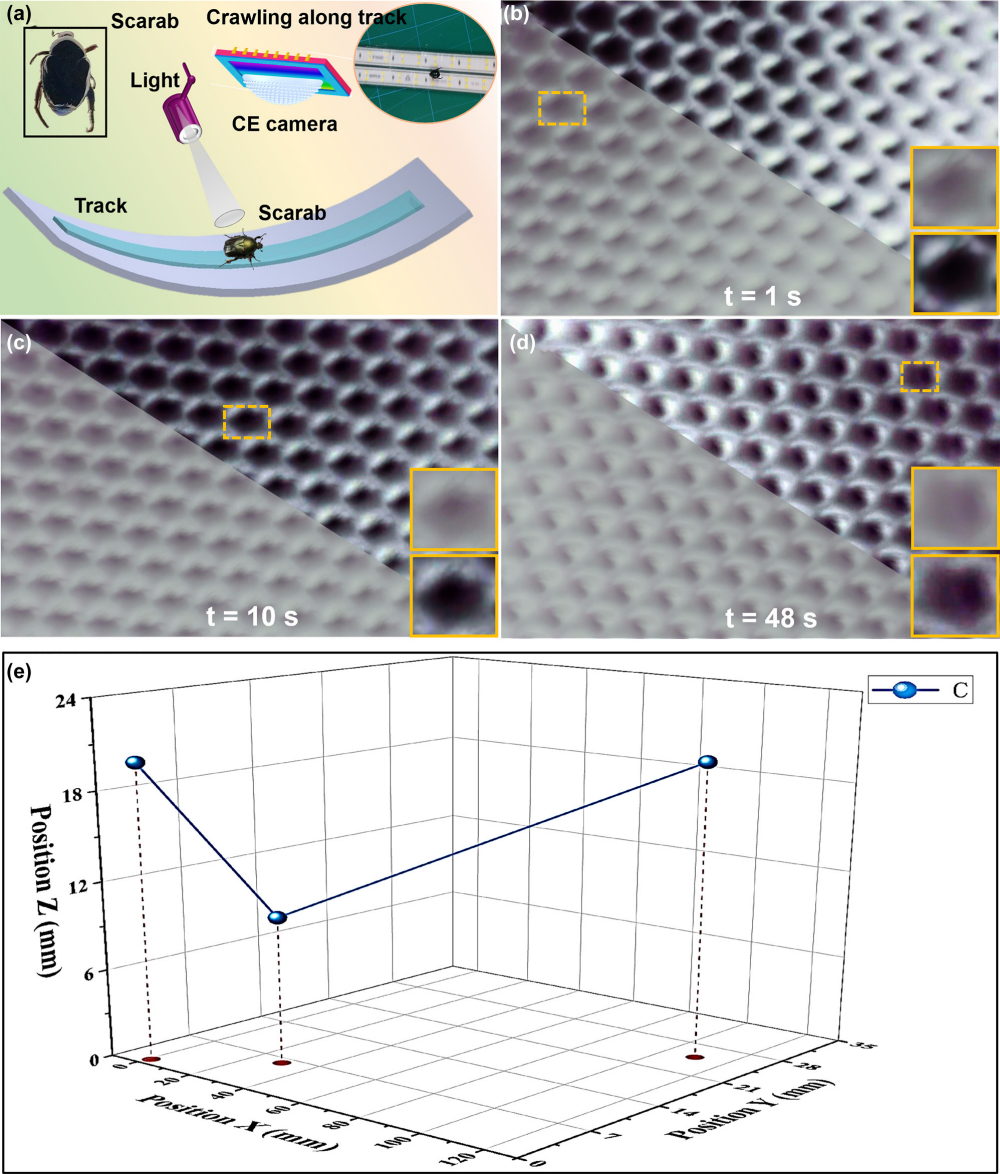
Recorded moving trajectory at different moments using proposed CE camera for a scarab. (a) A three-dimensional curved track covered with LED light strips. (b–d) Recorded moving trajectory for scarab at different moments. (e) Three-dimensional moving trajectory reconstruction.

The fabricated computational-CE camera has competitive advantage in the application *in vivo* moving trajectory reconstruction. Because insect CE vision is very sensitive to moving objects and has good perception of objects with depth, it is able to provide immediate and effective feedback. Inspired by the insect CE feature, our computational compound eye camera can perceive the position of moving objects in the entire three-dimensional space. To assess the ability in this application, a three-dimensional curved track covered with LED light strips is established, as illustrated in Figure 8(a). A live scarab crawls on a paved track, whose moving trajectory can be recorded using this computational-CE camera. Figure 8(b)–(d) recorded the moving trajectory at different moments using our proposed CE camera for a scarab. The comparison results at 1/10/48 s for the moving live scarab are illustrated in Figure 8(b)–(d), here lower left region represents the raw image without multi-branch reconstruction, and the upper right region represents the reconstructed image with a multi-branch model. The labeled-yellow regions demonstrate enlarged details. At the 1st and 10th seconds, the reconstructed image recovered feet from the degraded image, such as how many (2) feet are exactly displayed next to the torso. From the 1st second to the 48th second, to our findings, we also find that the computational-CE camera first captures the head, then the torso, and finally the buttocks of the scarab if we focus on the upper right region.

Unlike a traditional camera that can only determine the object’s distance with known true size of the object, the proposed CE camera enables three-dimensional moving trajectory reconstruction based on the principle of multi-eye vision. When we observe the target objects using the proposed CE camera, ommatidia with different orientations can image the same target from different angles. The reconstruction steps of the scarab’s movement trajectory are as follows: firstly, the proposed compound eye camera is placed in a fixed position and the camera parameters are calibrated. Secondly, the speeded up robust features (SURF) [33] algorithm is used to extract the feature points at different viewing angles of adjacent images. Furthermore, the extracted feature points are effectively matched, and finally, the three-dimensional spatial position of the scarab is measured by triangulation method according to the camera parameters and matching relationship. Based on the recorded information, the moving trajectory reconstruction is reconstructed in Figure 8(e).

### 3.9 Computational-CE imaging with distance regulation

**Figure 9:**
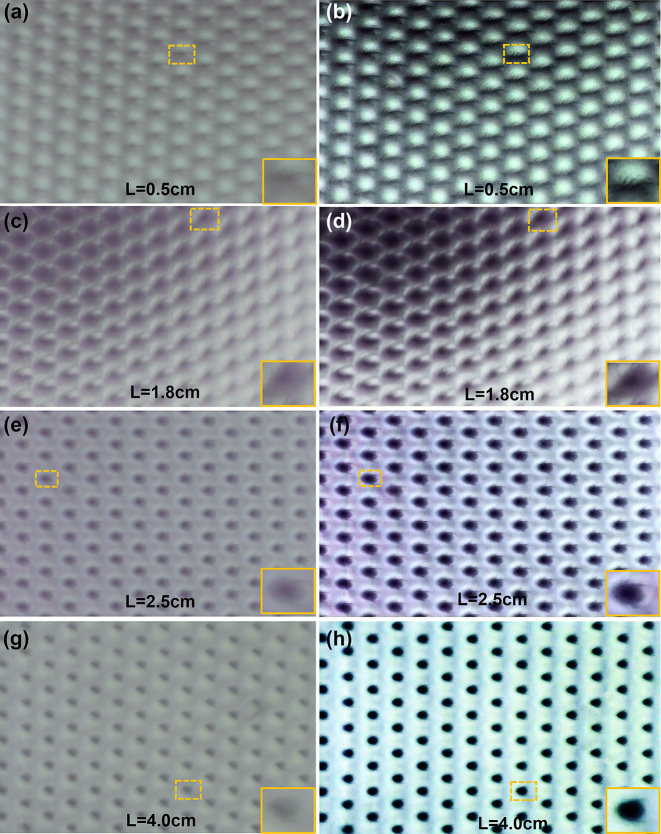
Comparison results based on distance regulation. (a, c, e, g) Left: results without multi-branch reconstruction. (b, d, f, i) Right: multi-branch reconstruction based on the distance regulation.

The comparison results with distance regulation or without it are illustrated in Figure 9(a)–(h). Due to the defocusing blur caused by imperfect imaging, the image details in the left images (a, c, e and g) are blurred, which is unrecognizable. As a comparison, the image details in the right images (b, d, f and h) are significantly recorded. Such as how many fuzzes (3) are exactly located on the foot at 0.5 cm (Figure 9(b)), and such as how many feet (3) are exactly bare on the torso at 2.5 cm (Figure 9(f)), and so on. The experimental result illustrates the multi-branch-regulation reconstruction makes image quality restoration possible at different distances.

### 3.10 Measurement of FOV

In this study, the experimental setup illustrated in Figure 8(a) is used to measure the FOV of our proposed CE camera. Here, the scale is labeled (1 cm). Notably, we can measure the FOV of this computational-CE camera when the entire target object fills the entire acquired image of the camera at a suitable location. As an example, the scarab illustrated in Figure 9(d) fills exactly the entire image, which proves that the FOV can be measured at this time. Based on the known size of the target object *d* is 2.3 cm and the object distance *L* is 1.8 cm, therefore, so the FOV *θ* value can be calculated to be 102° according to the following Equation (7):

(7) $$\theta =2\arctan\left(\frac{d}{2L}\right)$$

## 4 Conclusions

The topic of integrated compound eyes is a hot topic that has attracted much attention. To overcome the defocusing problem, a deep learning architecture with distance regulation is first proposed to achieve wide-range-clear imaging, without any hardware or specialized front-end design, which greatly reduces system complexity and size. Here we describe the high-resolution computational-CE camera with 271 ommatidia, with a weight of 5.4 g, an area of 3 × 3 cm^2^ and 5-mm thickness, which achieves compatibility and integration of CE with commercial CMOS. However, the performance of the system is expected to be further improved, the potential ways to further improve the performance include designing multi-layer compound eyes, more robust reconstruction algorithms and so on. In addition, sensor selection is also one of the methods. The compact camera has promising applications in integrated fields such as medical endoscopy, panoramic imaging and vision robotics.
